# Fabrication of Colon-Targeted Delivery System of Astaxanthin Based on Bacteroides-Dependent Biodegradation Strategy and Its Role in Ameliorating DSS-Induced Colitis in Mice

**DOI:** 10.3390/foods15101675

**Published:** 2026-05-11

**Authors:** Weiyun Zheng, Shugang Li, Yuxin Xu, Shuang Song, Chunqing Ai

**Affiliations:** 1 School of Agronomy and Life Sciences, Shanxi Datong University, Datong 037009, China; 2 School of Food Science and Technology, National Engineering Research Center of Seafood, Dalian Polytechnic University, Dalian 116034, China; lishugang688@163.com (S.L.); xuyuxin1163@163.com (Y.X.); songs1008@163.com (S.S.)

**Keywords:** astaxanthin, colon-targeted delivery, intestinal diseases, *Bacteroides* fermentation

## Abstract

Astaxanthin (Ax) is a potent antioxidant, yet its poor water solubility and instability limit its application. While alginate-Ca encapsulation protects Ax during digestion, its release in the colon is often inefficient. This study aims to optimize colon-targeted delivery by integrating inulin and fucoidan, which respond selectively to *Bacteroides*-mediated fermentation. A novel delivery system was developed using Ax-containing particles formulated in an alginate–inulin–fucoidan matrix (Ax-Mix), with most particles ranging from 2 to 20 μm. In vitro results showed that the incorporation of inulin and fucoidan enhanced Ax release in alginate-Ca (Ax-Alg) through *Bacteroides* fermentation. Ax-Mix exhibited robust structural integrity under varying pH, thermal, and ionic conditions. Ax-Mix remained intact through the oral cavity, stomach, and small intestine, but disintegrated in the colon, triggering Ax release. Ax-Mix alleviated colitis in mice, characterized by increased weight gain and colon length and reduced disease activity index, tissue damage, and oxidative stress. Ax-Mix reshaped the gut microbiota by increasing microbial diversity and enriching beneficial taxa linked to colitis improvement. These alterations resulted in increased propionate and butyrate production. Compared to Ax-Alg, Ax-Mix exhibited superior therapeutic effects on colitis, though the underlying mechanisms require further investigation. This study presents a promising strategy for microbiota-targeted delivery of active substances.

## 1. Introduction

Inflammatory bowel disease (IBD), including Crohn’s disease and ulcerative colitis, is a chronic inflammatory condition of the gastrointestinal tract [1]. Its rising incidence rate has emerged as a global health challenge due to its long-term impact on healthcare systems and patients’ quality of life [2]. The pathogenesis of IBD involves multiple factors, such as gut barrier dysfunction, immune dysregulation, genetic predisposition, and gut microbiota dysbiosis [3]. Current treatments primarily rely on pharmacological interventions such as corticosteroids, immunosuppressants, and surgical intervention [4]. However, these therapies often produce incomplete remission and side effects such as abdominal pain and diarrhea [5]. These challenges highlight the need for safer and more effective therapies targeting the underlying mechanism of IBD.

Natural bioactive compounds, e.g., astaxanthin (Ax) and resveratrol, have emerged as promising alternatives for IBD treatment [6]. Ax, a xanthophyll carotenoid, possesses potent antioxidant and anti-inflammatory properties, making it a promising candidate for inflammation-related diseases [7]. Ax exhibits exceptional free-radical-scavenging capacity, ranking it among the most effective natural antioxidants [8]. Studies have shown that Ax ameliorates (Dextran Sulfate Sodium) DSS-induced colitis by modulating MAPK and NF-κB signaling pathways [9], leading to reduced disease activity index (DAI), diminished inflammation, and improved intestinal barrier integrity [10]. These results imply that Ax has potential as a therapeutic agent for IBD treatment [11]. However, its clinical translation remains hindered by formulation-related challenges—including environmental instability (i.e., susceptibility to thermal, photochemical, acidic, and oxidative degradation), poor aqueous solubility, and low oral bioavailability [12,13,14,15].

Encapsulation technologies provide advanced strategies for enhanced protection, improved stability, and controlled release of bioactive compounds [16]. Among various technologies, alginate-based delivery systems have gained significant attention due to their rapid gelation, non-toxicity, and biocompatibility [17], making them attractive for oral administration [18]. Due to their ability to respond to gastrointestinal pH changes and microbial activity, alginate carriers are commonly used for colon-targeted delivery applications [19,20]. For instance, Ax-loaded alginate microparticles (Ax-Alg) have shown improved protection against digestive degradation and superior therapeutic effects against colitis compared to free Ax [16]. Despite these benefits, the release rate of Ax from Ax-Alg is limited by the physicochemical nature of Alg. To address this, co-encapsulation with proteins and polysaccharides has been investigated. Notably, fucoidan, a sulfated polysaccharide derived from brown algae, possesses anti-inflammatory and immunomodulatory activities [21], whereas inulin, a prebiotic fructan, promotes beneficial gut microbiota and enhances colonic fermentation [21]. Incorporating these components into alginate-based delivery systems enables synergistic modulation of carrier degradation kinetics and Ax release profiles in response to colonic microbial enzymatic activity. However, these modifications are often guided by empirical observations rather than a clear mechanistic understanding. This highlights the need for rational strategies that align release profiles with microbial activity in the gut.

In this study, a colon-targeted microparticle (Ax-Mix) was designed to enhance the colonic release of Ax by leveraging the selective fermentation capacities of Bacteroides strains toward alginate, fucoidan, and inulin. The formulation was characterized in vitro for its particle size distribution, thermal and pH stability, and resistance to ionic stress. We evaluated its gastrointestinal release profiles using in vitro digestion models. Then, the therapeutic efficacy of Ax-Mix was further validated in DSS-induced colitis mice. This study aims to establish Ax-Mix as a promising microbiota-responsive delivery system with high potential for managing IBD.

## 2. Materials and Methods

### 2.1. Materials and Chemicals

Ax (purity > 98%) was obtained from Xi’an Realin Biotechnology Co., Ltd. (Xi’an, China). Alg was purchased from Qingdao Mingyue Seaweed Group Co., Ltd. (Qingdao, China). Lecithin was provided by Aladdin (Shanghai, China). DSS (Mw: 36–50 kDa, purity > 98%) was from MP Biomedicals LLC (Santa Ana, CA, USA). Myeloperoxidase (MPO), glutathione peroxidase (GSH-Px), malondialdehyde (MDA), total superoxide dismutase (T-SOD), inducible nitric oxide synthase (iNOS), and catalase (CAT) assay kits were supplied by Jiancheng Bioengineering Institute (Nanjing, China).

### 2.2. Bacteroides Strains Culture

*Bacteroides* strains were obtained from the National Engineering Research Center of Seafood, Dalian, Liaoning, China. These strains were cultured in brain heart infusion (BHI) medium (Hope Bio-Technology Co., Ltd., Qingdao, China) and incubated anaerobically in an Electrotek Workstation (AW500SG/TG, West Yorkshire, UK) at 37 °C for 24 h.

### 2.3. Utilization Analysis of Polysaccharides by Bacteroides Strains

After 24 h culture, *Bacteroides* strains were washed and resuspended in sterile phosphate-buffered saline (PBS), then inoculated (1% *v*/*v*) into the chemically defined medium (DM) medium (Table S1) containing 0.5% (*w*/*v*) fucoidan, alginate, or inulin as the sole carbon source. After incubation at 37 °C for 72 h, residual polysaccharides in the medium were quantified using the phenol–sulfuric acid method [22]. This assay relies on acid hydrolysis of polysaccharides to generate furfural derivatives, which subsequently condense with phenol to yield orange–yellow compounds; the absorbance measured at 490 nm is directly proportional to polysaccharide concentration.

### 2.4. Fabrication of Ax-Alg and Ax-Mix

**Figure 1:**
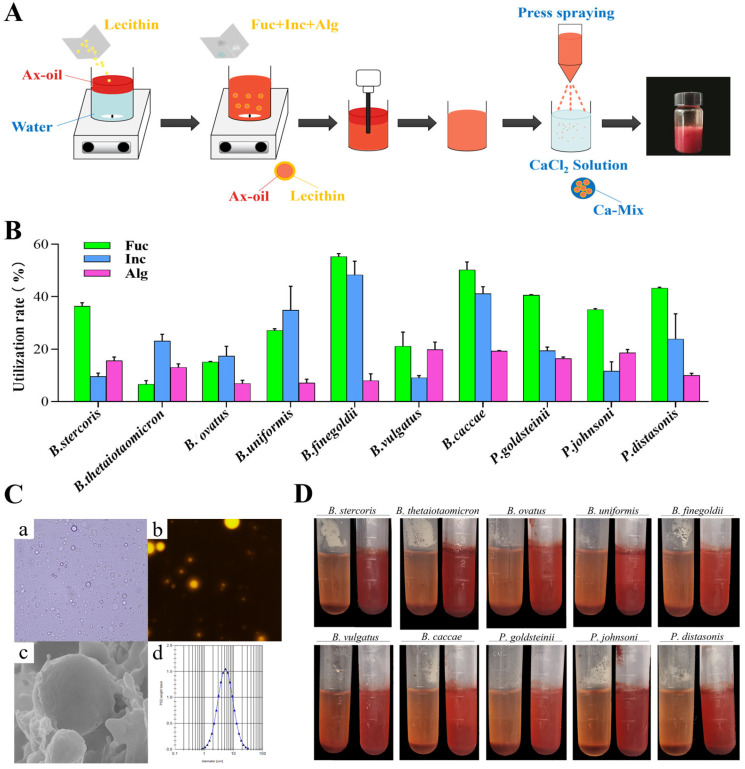
Fabrication and characteristic analysis of Ax-Mix. Flow diagram of Ax-Mix (**A**). Utilization rate of fucoidan, inulin, and Alg by Bacteroides strains (**B**). Characteristics analysis of Ax-Mix, including (**a**) optical microscope, (**b**) fluorescence microscope, (**c**) Cry-SEM, and (**d**) particle size distribution (**C**). Comparative analysis of Ax release in Ax-Alg (left) and Ax-Mix (right) in the in vitro fermentation model (**D**). Fuc: fucoidan; Inc: inulin; Alg: alginate-Ca.

Ax-Alg was prepared as previously described [16]. Briefly, Ax dissolved in olive oil (1%, *w*/*v*) and lecithin in water (5%, *w*/*v*) were emulsified (1:9) to form an oil-in-water emulsion, which was then mixed with alginate (3%, *w*/*v*) and sprayed into 7% CaCl_2_ solution via high-pressure spraying to obtain Ax-Alg. Ax-Mix was fabricated as shown in Figure 1A. Ax was dissolved in absolute ethanol, stirred at 4 °C in the dark for 2 h, and centrifuged (5000 rpm, 4 °C, 15 min). The supernatant was evaporated to obtain the Ax oil phase. The aqueous phase was prepared by dispersing 5% (*w*/*v*) lecithin in water. Oil and water phases were mixed at a 1:9 (*v*/*v*) ratio and homogenized (8000 rpm, 5 min), followed by high-pressure homogenization (540 MPa, 5 cycles) to form an oil-in-water emulsion. The emulsion was fortified with 1% (*w*/*v*) alginate, fucoidan, and inulin, then sprayed into 7% (*w*/*v*) CaCl_2_ solution under stirring for 30 min to yield Ax-Mix particles.

### 2.5. Encapsulation Efficiency of Ax-Alg

The encapsulation efficiency (EE) of Ax was determined by measuring the absorbance of the supernatant at 483 nm after centrifugation at 5000 rpm for 10 min. The EE was calculated as:

$$EE\left(\%\right)=\frac{Wt-Ws}{Wt}\times 100\%$$

where *Wt* is the total amount of Ax initially added, and *Ws* is the amount of unencapsulated Ax in the supernatant.

### 2.6. Morphological Analysis of Ax-Mix

The morphology of Ax-Mix was analyzed using our previously established method [16]. Briefly, the particle shape and size were measured via optical microscopy (BX53, Olympus Corporation, Tokyo, Japan). The surface morphology was examined using Cryo-SEM (SU8010, Hitachi, Tokyo, Japan). The size distribution was analyzed with an Acoustic and Electroacoustic spectrometer (DT-1202, Dispersion Technology Inc., Bedford Hills, NY, USA). Encapsulation of Ax within Ax-Mix was confirmed using Nile red staining (0.1 mg/mL) and observed under a fluorescence microscope (Eclipse Ti-S, Nikon, Tokyo, Japan).

### 2.7. Stability Assessment of Ax-Mix

The stability of Ax-Mix was assessed under varying ionic strength, temperature, and pH conditions, following our previously established protocol [16]. The stability of Ax-Alg was evaluated under the following controlled conditions: pH (2.0, 7.0 [control], and 8.0), ionic strength (0.1 and 0.3 M NaCl, pH7.0), and temperature (45 °C and 60 °C). Each condition was applied for 6 h; all assays—except those assessing thermal stability—were performed at 25 °C.

### 2.8. Digestive Properties of Ax-Mix in Upper Digestive Models

Simulated digestive fluids—salivary, gastric, and intestinal—were prepared according to the following standardized protocols [23]. The digestive properties of Ax-Mix were analyzed using the previously validated in vitro method [24]. Specifically, Ax-Mix was added to each digestive juice and incubated at 37 °C for 6 h (mouth and stomach phases) or 24 h (small intestine phase). Following incubation, Ax-Mix particles and corresponding supernatants were collected for further analysis.

### 2.9. Digestion Properties of Ax-Mix in In Vitro Colon Model

The digestion properties of Ax-Mix in the colon were evaluated using an in vitro fermentation model, as described in our previous study [25]. A microbial suspension from healthy human feces was added to gut microbiota medium containing Ax-Alg at a 1:9 ratio, incubated at 37 °C for 72 h, and then separated into Ax-Alg and supernatant for further analysis, with untreated Ax-Alg serving as the control.

### 2.10. Mice Experiment

Male BALB/c mice (specific pathogen-free, 6 weeks old, 20 ± 2 g) were purchased from Changsheng Biotechnology Co., Ltd. (Benxi, China) and acclimated to standard conditions (25 ± 3°C, 55 ± 5% humidity, 12 h light/dark cycle) for one week. Animal experiments were performed following the National Institutes of Health’s Guidelines for the Care and Use of Laboratory Animals and approved by the Animal Ethics Committee of Dalian Polytechnic University (No. DLPU2024DT005).

Mice were randomly assigned to five groups (*n* = 7/group): normal control (CTL), DSS, Ax, Ax-Alg, and Ax-Mix groups. Mice in the CTL group received distilled water daily via oral gavage, whereas those in the DSS, Ax, Ax-Alg, and Ax-Mix groups were administered 1.5% DSS (*w*/*v*) solution using the same route and frequency for 9 weeks. Concurrently, mice in the Ax, Ax-Alg, and Ax-Mix groups were administered with Ax, Ax-Alg and Ax-Mix (30 ppm Ax in 200 μL) via oral gavage. Disease activity index (DAI) was recorded weekly as previously described [26], based on body weight loss, stool consistency, and fecal occult blood. At the end of the experiment, mice were anesthetized with isoflurane and euthanized. Biological samples, including serum, colon, and cecum, were collected for further analysis.

### 2.11. Histopathology Analysis

Colon tissues were fixed in 4% paraformaldehyde, embedded in paraffin, sectioned, and stained with hematoxylin and eosin (HE) and Alcian blue-periodic acid-Schiff (AB-PAS). Histopathological changes were assessed following the established method [4], with colon tissue sections evaluated in a blinded manner under light microscopy for inflammation extent (0–4), inflammation severity (0–4), and crypt damage (0–4).

### 2.12. Biochemical Analysis

Colon tissues were homogenized in ice-cold PBS solution at a 1:9 (*w*/*v*) ratio. Following centrifugation under conditions specified in the respective manufacturer’s instructions, supernatants were assayed for MPO, iNOS, CAT, GSH-Px, T-SOD, and MDA using commercially available assay kits.

### 2.13. Analysis of Fecal Microbiota

Fecal samples were subjected to 16S rRNA gene (v3–v4 regions) sequencing, performed by Biomarker Technology Co., Ltd. (Beijing, China) as described in our previous study. Microbial α-diversity was assessed using the Shannon index—which integrates species richness and evenness—while β-diversity was evaluated by principal coordinate analysis (PCoA) of weighted UniFrac distances to visualize similarities in microbiota communities across groups [27]. Hierarchical clustering using the unweighted pair group method with arithmetic means (UPGMA) was performed to assess similarities between groups.

### 2.14. Measurement of Short Chain Fatty Acids (SCFAs)

SCFAs levels in cecal contents were measured using gas chromatography (Agilent 7890B, Agilent Technologies, Santa Clara, CA, USA), as described in our previous study [28]. Briefly, 0.1 g of fecal sample was homogenized in saturated NaCl solution, acidified with HCl to pH < 2, extracted twice with diethyl ether, and analyzed using an HP-INNOWAX capillary column (30 m × 0.25 mm × 0.25 μm, Agilent Technologies, Santa Clara, CA, USA) under a temperature program ramping from 100 °C to 200 °C at 5 °C/min.

Under Article 32, Paragraph 3 of the *Measures for Ethical Review of Life Science and Medical Research Involving Human Subjects* (National Health Commission, Guo Wei Ke Jiao Fa [2023] No. 4), fecal specimens are exempt from ethics review.

### 2.15. Statistical Analysis

All data are presented as means ± standard error of the mean (SEM). Statistical analysis was performed using GraphPad Prism 9.0 software. One-way analysis of variance, followed by Tukey’s multiple comparison test, was used to evaluate statistical differences between groups. A *p*-value < 0.05 is considered statistically significant. * *p* < 0.05, ** *p* < 0.01, *** *p* < 0.005, and **** *p* < 0.001.

## 3. Results

### 3.1. Effect of Polysaccharides Utilization by Bacteroides on Ax Release from Ax-Mix

To determine how polysaccharide composition affects the release of Ax from Alg-based systems, the ability of *Bacteroides* species to utilize fucoidan, inulin, and Alg was evaluated in an in vitro model (Figure 1B). It showed that *B. stercoris*, *P. goldsteinii*, *P. johnsonii*, and *P. distasonis* had a greater capacity to utilize fucoidan than inulin and Alg, while *B. finegoldii*, *B. uniformis*, *B. ovatus*, and *B. caccae* more efficiently metabolized fucoidan and inulin. Additionally, *B. thetaiotaomicron* showed the highest response to inulin, while *B. vulgatus* utilized fucoidan and Alg at similar rates. This species-specific utilization pattern implies that integrating fermentable polysaccharides into the Alg matrix may enhance Ax release in the colon.

Ax-Mix delivery system was fabricated through emulsification, followed by high-pressure spraying and ionic crosslinking with Ca. The EE was determined to be 74.57%. Morphological analysis using optical and fluorescence microscopes confirmed the successful embedding of Ax-loaded O/W emulsion within Ax-Mix (Figure 1C). Cryo-SEM revealed that Ax-Mix formed discrete, spherical microparticles with diameters predominantly between 2 and 20 μm. Notably, the addition of fucoidan and inulin did not interfere with the Ca-mediated gelation of Alg, maintaining structural integrity. When exposed to *Bacteroides* in vitro, Ax-Mix exhibited enhanced breakdown and Ax release relative to Ax-Alg particles (Figure 1D). These results support the rationale for designing compound polysaccharide matrices to optimize microbial-triggered Ax release, with potential applications in IBD and other intestinal disorders.

### 3.2. Stability Analysis of Ax-Mix Under Different Environmental Conditions

**Figure 2:**
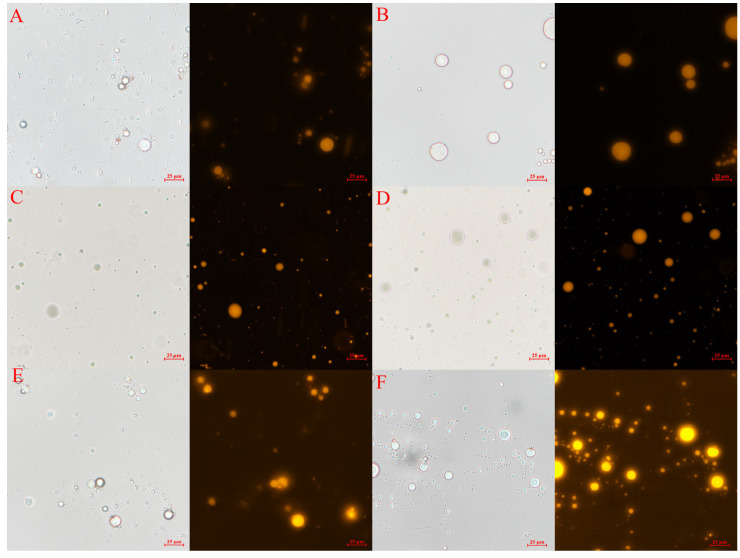
Stability analysis of Ax-Mix under different conditions: 0.1 M NaCl (**A**), 0.3 M NaCl (**B**), pH 2.0 (**C**), pH 8.0 (**D**), 45 °C (**E**), and 60 °C (**F**). Optical microscope (left) and fluorescence microscope (right).

To assess its stability, Ax-Mix was subjected to simulated environmental stressors, including ionic strength, pH variation, and temperature shifts (Figure 2). When exposed to 0.1 M and 0.3 M NaCl and pH 2.0 and 8.0, Ax-Mix retained its spherical morphology without visible structural disruptions. Fluorescence microscopy revealed no detectable release of Ax-loaded O/W emulsion (Figure 2A–D). This suggests that the ionic crosslinks between Alg and Ca remained intact under these conditions. Under thermal stress, Ax-Mix maintained overall structural integrity at 45 °C and 60 °C, though a gradual increase in background fluorescence was observed, especially at 60 °C (Figure 2E,F). This suggests that heat partially compromised the gel matrix, resulting in the leakage of Ax-loaded emulsion. These indicate that Ax-Mix exhibits high stability under physiologically relevant ionic and pH conditions, along with moderate heat tolerance, which support its viability for food applications.

### 3.3. Digestive Characteristics of Ax-Mix in the In Vitro Models

**Figure 3:**
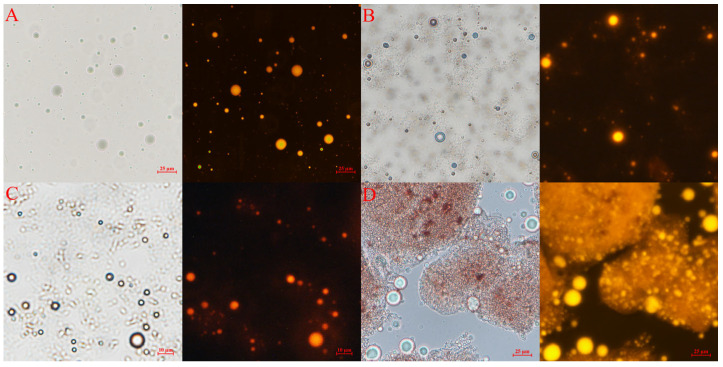
Digestive characteristics of Ax-Mix in in vitro models. Representative images from the mouth (**A**), stomach (**B**), small intestine (**C**), and colon (**D**) models. Optical microscope (left) and fluorescence microscope (right).

To assess the suitability of Ax-Mix for targeted colon delivery, its digestive profiles were evaluated in simulated in vitro models (Figure 3). Ax-Mix maintained its structural integrity after exposure to simulated oral, gastric, and small intestinal juices, with no significant changes or fluorescence signals observed (Figure 3A–C). This suggests that Alg-inulin–fucoidan matrix effectively shields Ax from enzymatic degradation and pH fluctuations in the upper digestive tract. However, significant fluorescence from Ax-loaded emulsion was detected in the aqueous phase following microbial fermentation (Figure 3D). These results indicate that microbial enzymatic activity disrupts the matrix, enabling Ax-Mix to resist gastric and small intestinal digestion and facilitating targeted colonic release of Ax—thereby positioning it as a promising platform for site-specific delivery of bioactive compounds in IBD management.

### 3.4. Ax-Mix Effectively Alleviated the Symptoms of Colitis

**Figure 4:**
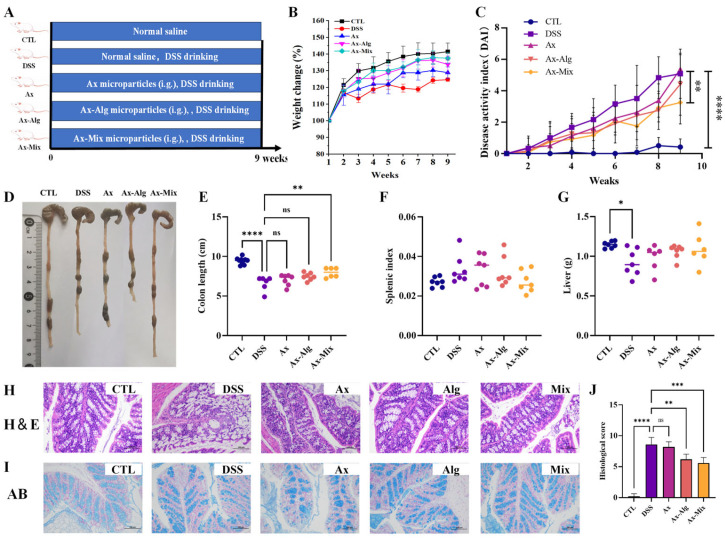
Effect of Ax-Mix on colitis symptoms in DSS-treated mice. Experimental design (**A**), body weight change (**B**), DAI (**C**), colon appearance (**D**), colon length (**E**), spleen index (**F**), and live weight (**G**). Histology analysis of colon tissues based on HE (**H**) and AB (**I**) staining. Histology scores (**J**). All data are presented as mean ± SEM. *n* = 5–6. * *p* < 0.05, ** *p* < 0.01, *** *p* < 0.001, and **** *p* < 0.0001.

To evaluate the anti-inflammatory effects of Ax-Mix, a DSS-induced colitis model was established in mice (Figure 4A). The DSS group exhibited significant weight loss and increased DAI scores compared to the CTL group (Figure 4B,C). Ax-Mix significantly mitigated weight loss and reduced DAI more prominently than Ax-Alg. Macroscopic analysis revealed severe colon shortening in DSS-treated mice, a common marker of colonic inflammation (Figure 4D,E). Ax-Mix markedly restored colon length, whereas no obvious change was observed in the Ax-Alg group. No differences in the spleen index and liver weight were observed across groups (Figure 4F,G). The histological examination revealed severe colitis symptoms in the DSS group, characterized by goblet cell loss, inflammatory infiltration, crypt disruption, and mucin thinning (Figure 4H,I). Ax-Alg and Ax-Mix mitigated these tissue changes, resulting in lower histology scores, whereas Ax showed limited improvements in tissue damage (Figure 4J). Taken together, Ax-Mix offered the most comprehensive protection, supporting its potential as a superior colon-targeted therapeutic for IBD management.

### 3.5. Ax-Mix Attenuated Oxidative Stress in Colon Tissues

**Figure 5:**
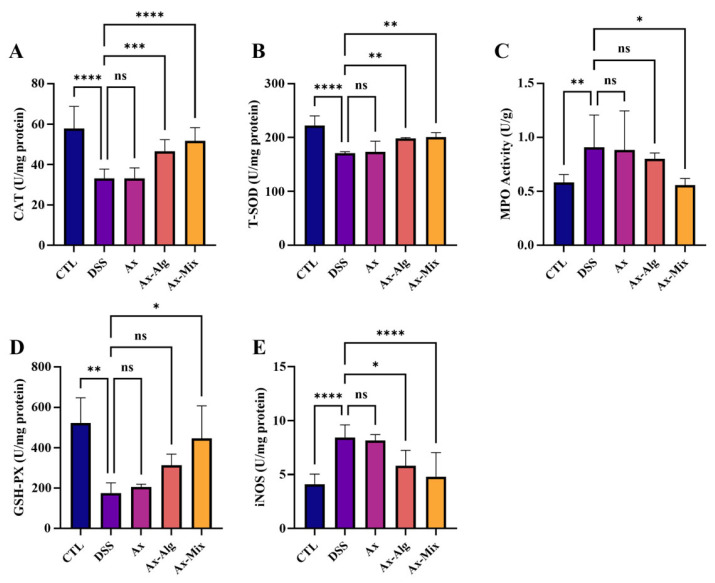
Inhibitory effect of Ax-Mix on oxidative stress in colon tissues. The levels of CAT (**A**), T-SOD (**B**), MPO (**C**), GSH-Px (**D**), and iNOS (**E**). All data are presented as mean ± SEM. *n* = 5. * *p* < 0.05, ** *p* < 0.01, *** *p* < 0.001, **** *p* < 0.0001.

As shown in Figure 5, the levels of CAT, T-SOD, and GSH-Px were significantly decreased in the DSS group compared to the CTL group, while iNOS and MPO levels were increased. In contrast, Ax treatment had no effect on these parameters. However, Ax-Mix significantly increased the levels of CAT, T-SOD, and GSH-Px and decreased iNOS and MPO levels, indicating an alleviation of oxidative stress. Notably, Ax-Alg also exerted an antioxidative effect, as evidenced by increases in CAT and T-SOD and a reduction in iNOS. This suggests that Ax-Mix more effectively enhances endogenous antioxidant defense, contributing to its superior therapeutic potential in managing IBD-related oxidative injury.

### 3.6. Ax-Mix Modulated Dysbiosis of Gut Microbiota in DSS-Treated Mice

**Figure 6:**
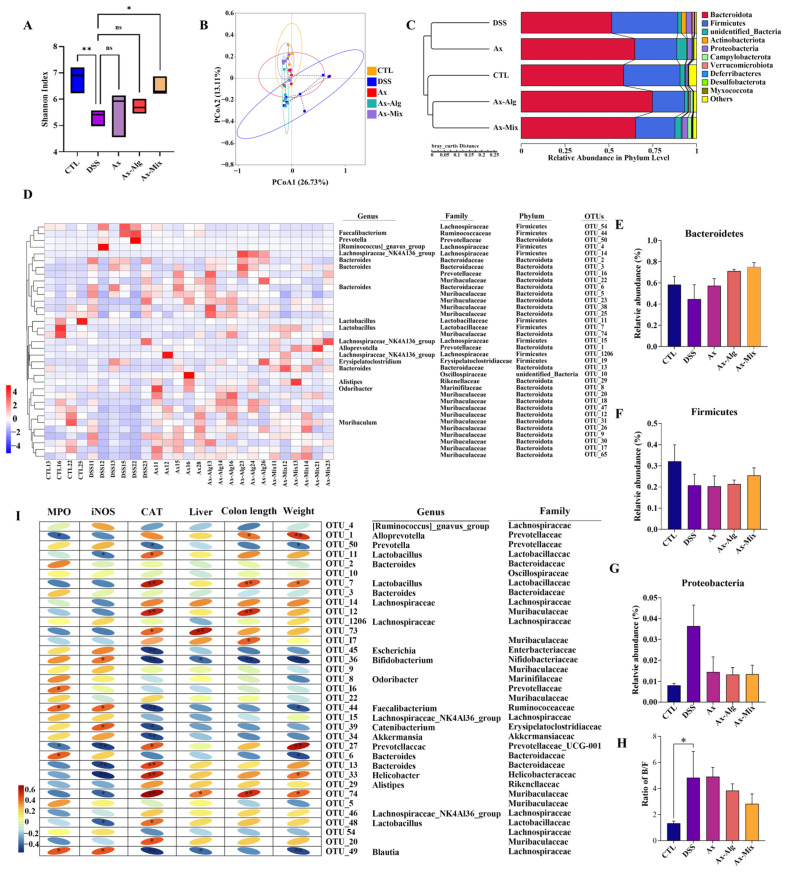
Effect of Ax-Mix on gut microbiota in the DSS-induced colitis mice. Shannon index (**A**), PCoA of the β-diversity (**B**), UPGMA (**C**), species abundance cluster heatmap (**D**), relative abundance of Bacteroidetes (**E**), Firmicutes (**F**) and Proteobacteria (**G**), correlation analysis of specific gut bacteria and inflammatory factors (**I**), ratio of Bacteroidetes/Firmicutes (**H**). All data are presented as mean ± SEM. *n* = 4–6. * *p* < 0.05, ** *p* < 0.01.

The effect of Ax-Mix on the gut microbiota was evaluated by sequencing. The Shannon index—a measure of microbial diversity from 16S rRNA data-was markedly reduced in the DSS group compared to the CTL group, whereas Ax-Mix increased the Shannon index, with no difference observed in the Ax and Ax-Alg groups (Figure 6A). It indicates that Ax-Mix restored microbiota diversity to a near-control level. PCoA revealed a clear separation between the CTL and DSS groups, while Ax, Ax-Alg, and Ax-Mix shifted the gut microbiota closer to the CTL group, with Ax-Mix showing the most pronounced effect (Figure 6B). UPGMA further showed that microbial profiles of the Ax-Alg and Ax-Mix groups were distinct from those of the DSS group (Figure 6C). These results suggest that Ax-Mix attenuates colitis at least partially via modulation of the gut microbiota.

As shown in Figure 6D, bacterial taxa that markedly altered between groups belonged to Bacteroidota and Firmicutes, involving various key families, such as Lachnospiraceae, Lactobacillaceae, Muribaculaceae, and Ruminococcaceae. Notably, Bacteroidota and Firmicutes remained unchanged across the groups, but Proteobacteria were significantly increased in the DSS group (Figure 6E–G). In contrast, Ax, Ax-Alg, and Ax-Mix had no effect on Bacteroidota and Firmicutes but decreased Proteobacteria (Figure 6E–G). At the family level, Prevotellaceae, Bacteroidaceae and Muribaculaceae were elevated in the DSS group, while Lachnospiraceae, Lactobacillaceae and Ruminococcaceae were reduced. In contrast, Prevotellaceae, Bacteroidaceae, Lactobacillaceae, and Ruminococcaceae were decreased in the Ax-Alg and Ax-Mix groups.

A Spearman correlation analysis was used to establish the correlation between key taxa and physiological markers (Figure 6I). The results showed that Lactobacillus, Muribaculaceae, and Prevotellaceae were positively correlated with CAT, colon length, and body weight. In addition, Helicobacteraceae and Prevotellaceae were negatively correlated with MPO and iNOS. These results showed that Ax-Mix modulates gut microbiota composition, suggesting a microbiota-dependent mechanism underpinning its therapeutic benefits in colitis.

### 3.7. Ax-Mix Promoted the Production of SCFAs in Colitis Mice

**Figure 7:**
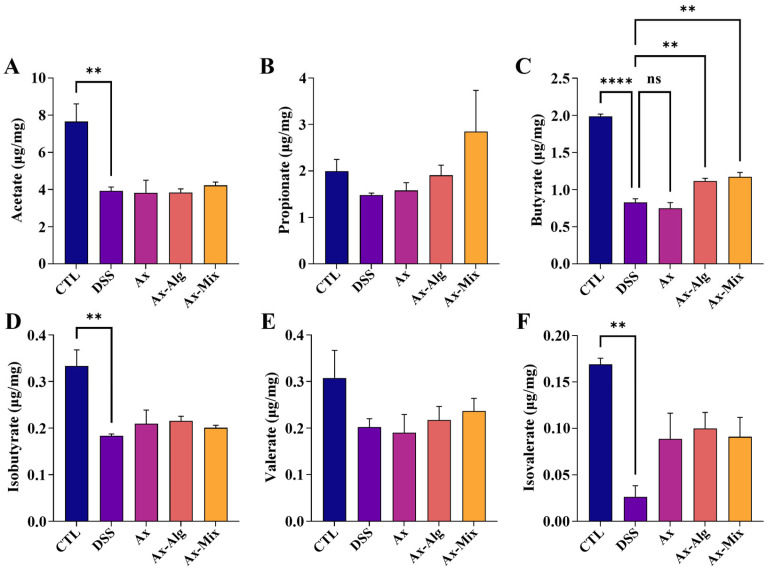
Effect of Ax-Mix on SCFAs levels in DSS-treated mice. The levels of acetate (**A**), propionate (**B**), butyrate (**C**), isobutyrate (**D**), valerate (**E**), and isovalerate (**F**). All data are presented as mean ± SEM. ** *p* < 0.01, **** *p* < 0.0001.

Compared to the CTL group, DSS treatment markedly reduced the levels of acetate, butyrate, isobutyrate, and isovalerate, whereas no significant differences were found in propionate and valerate (Figure 7). In contrast, Ax had no significant effect on the levels of SCFAs, except isovalerate, whereas Ax-Alg and Ax-Mix significantly increased the levels of butyrate and isovalerate. These results imply that Ax-Mix can alleviate colitis via SCFAs-mediated pathways.

## 4. Discussion

The emergence of colon-targeted delivery systems represents an advancement in the treatment of IBD, enabling localized therapeutic effects while minimizing systemic side effects [29]. This approach is particularly valuable given the chronic and relapsing nature of IBD. Recent studies suggest that integrating bioactive compounds into colon-targeted delivery systems can effectively overcome the limitations of current treatment [30]. In this study, a novel Ax-loaded colon-targeted delivery system was fabricated by embedding Ax in an optimized Alg-Ca system. Compared to conventional Ax-Alg, Ax-Mix exhibited superior efficacy in mitigating colitis, due to improved release kinetics and microbiota-responsive degradation.

Polysaccharides offer a safe and effective strategy for colon-targeted delivery due to their microbiota-responsiveness [31]. Carotenoid bioavailability has been shown to be significantly enhanced via polysaccharide encapsulation [32]. Some polysaccharides, e.g., guar gum, inulin, and chitosan, have been extensively explored for colon-targeted delivery of small molecules [33,34,35]. They are characterized by biodegradability, non-toxicity, and stability, making them well-suited for targeted delivery to the colon [36]. Here, Alg, inulin, and fucoidan—each fermentable by *Bacteroides*—were used to construct a delivery system that remains intact in the upper GI tract but degrades in the colon. In vitro fermentation results showed that fucoidan and inulin were more readily utilized by *Bacteroides* strains than Alg alone. This enhanced fermentability likely contributed to a more effective release of Ax from Ax-Mix, enabling its targeted therapeutic effect. This targeted responsiveness to bacterial activity not only supports colon delivery but also represents a strategic departure from pH-triggered systems, which are inconsistent due to inter-individual variability in intestinal pH.

Ax-Mix maintained structural integrity across a range of environmental conditions, including variations in pH, ionic strength, and temperature. Recent studies have shown that Alg forms heat-stable gels; however, heated Alg gels may become softer, but they maintain structural integrity [37,38]. Minimal release of Ax-loaded emulsions after heating suggests that leakage may occur from the surface or outer layers of the matrix. Additionally, the resistance of Ax-Mix to degradation in the upper GI tract is essential for ensuring Ax reaches the colon intact. This is consistent with the fact that Alg-based systems can withstand GI transit and delay its release until microbial fermentation occurs [16]. Under acidic conditions, Alg particles shrink and swell, leading to increased pore sizes, yet the overall structure of Ax-Mix remained intact [39]. These results indicate that Ax-Mix maintains its structural integrity under these conditions, highlighting its potential applications in the food and medical fields.

The therapeutic effect of Ax-Mix on colitis was further assessed in a mouse model. Compared to Ax and Ax-Alg, Ax-Mix more effectively attenuated symptoms of colitis, such as DAI, body weight, and colon length. These differences may stem directly from improved Ax bioavailability in the colon. Moreover, Ax-Mix significantly attenuated oxidative stress in the colon tissues, as indicated by increased key antioxidant enzymes (CAT, SOD, GSH-Px) and reduced oxidative markers. Oxidative stress is a potential pathogenic factor in UC, mainly due to the imbalance between reactive oxygen species (ROS) production and antioxidant defenses [40]. ROS can activate signaling pathways that promote inflammation [41]. Previous studies showed that Ax attenuated oxidative damage in the burn model by modulating antioxidant enzyme levels [42]. Additionally, Ax alleviated mucosal damage in a necrotizing enterocolitis model, which was associated with increased SOD and GSH [43]. In this study, Ax-Mix significantly increased CAT, SOD, and GSH-Px levels while reducing iNOS levels, suggesting enhanced antioxidant defense. Notably, Ax-Mix outperformed Ax-Alg in alleviating oxidative stress. This can be attributed to increased Ax release in the colon and enhanced microbial fermentation of matrix components, which may generate additional anti-inflammatory or antioxidant metabolites.

The gut microbiota plays a crucial role in host metabolism and immune regulation. Dysbiosis has been implicated in various inflammatory diseases, including IBD [44]. Increasing evidence has shown that the gut microbiota diversity and composition are significantly reduced in IBD mice [45]. In this study, Ax-Mix altered the composition of gut microbiota, effectively reversing the reduction in microbial diversity. While all the treatments exerted some regulatory effect, Ax-Mix most closely approximated the microbiota profile of health controls. Notably, Ax-Mix reduced the relative abundance of Proteobacteria—a phylum often increased in colitis and linked to pro-inflammatory activity—and increased the abundance of beneficial taxa such as Lactobacillaceae and Ruminococcaceae. This microbiota shift was accompanied by elevated levels of SCFAs, particularly butyrate, which is known to enhance colonic barrier function and regulate immune responses [46]. The interplay between Ax-Mix, microbiota modulation, and host physiology is central to its health-promoting role. The microbial degradation of inulin and fucoidan likely fosters the growth of SCFAs-producing bacteria, which in turn contributes to local immune regulation and oxidative stress mitigation. Therefore, Ax-Mix functions not only as a delivery vehicle but also as a prebiotic agent, creating a feedback loop that supports intestinal homeostasis.

## 5. Conclusions

This study fabricated a novel colon-targeted delivery system that withstands upper GI conditions and releases Ax in the colon. This system exerts a clear therapeutic benefit in colitis mice, as indicated by increased body weight and colon length and attenuated oxidative stress and tissue damage, outperforming the effect of Ax-Alg. These effects may be attributed to increased colonic release of Ax and modulation of gut microbiota and metabolites, such as SCFAs. It suggests synergy between the prebiotic matrix and Ax, supporting Ax-Mix as a safe, simple, and cost-effective candidate for functional foods or adjunct IBD therapy. Nevertheless, some limitations still need to be further addressed, e.g., Ax release and pharmacokinetics in vivo, long-term stability and manufacturability, and efficacy during long-term use.

## Data Availability

The original contributions presented in the study are included in the article/Supplementary Material, further inquiries can be directed to the corresponding authors.

## Supplementary Materials

The following supporting information can be downloaded at https://www.mdpi.com/article/10.3390/foods15101675/s1, Table S1. DM medium formulae.
